# BQsupports: systematic assessment of the support and novelty of new biomedical associations

**DOI:** 10.1093/bioinformatics/btad581

**Published:** 2023-09-19

**Authors:** Adrià Fernández-Torras, Martina Locatelli, Martino Bertoni, Patrick Aloy

**Affiliations:** Institute for Research in Biomedicine (IRB Barcelona), The Barcelona Institute of Science and Technology, Barcelona, Catalonia, Spain; Institute for Research in Biomedicine (IRB Barcelona), The Barcelona Institute of Science and Technology, Barcelona, Catalonia, Spain; Institute for Research in Biomedicine (IRB Barcelona), The Barcelona Institute of Science and Technology, Barcelona, Catalonia, Spain; Institute for Research in Biomedicine (IRB Barcelona), The Barcelona Institute of Science and Technology, Barcelona, Catalonia, Spain; Institució Catalana de Recerca i Estudis Avançats (ICREA), Barcelona, Catalonia, Spain

## Abstract

**Motivation:**

Living a Big Data era in Biomedicine, there is an unmet need to systematically assess experimental observations in the context of available information. This assessment would offer a means for a comprehensive and robust validation of biomedical data results and provide an initial estimate of the potential novelty of the findings.

**Results:**

Here we present BQsupports, a web-based tool built upon the Bioteque biomedical descriptors that systematically analyzes and quantifies the current support to a given set of observations. The tool relies on over 1000 distinct types of biomedical descriptors, covering over 11 different biological and chemical entities, including genes, cell lines, diseases, and small molecules. By exploring hundreds of descriptors, BQsupports provide support scores for each observation across a wide variety of biomedical contexts. These scores are then aggregated to summarize the biomedical support of the assessed dataset as a whole. Finally, the BQsupports also suggests predictive features of the given dataset, which can be exploited in downstream machine learning applications.

**Availability and implementation:**

The web application and underlying data are available online (https://bqsupports.irbbarcelona.org).

## 1 Introduction

Since the popularization of high-throughput experiments to obtain more unbiased and comprehensive description of biology, many initiatives have massively gathered data from biological systems (Evans 2000). Initial efforts relied on model organism screenings to uncover protein–protein interactions (PPIs) (Rual *et al.* 2005) and gene co-expression patterns (Obayashi *et al.* 2008). Concurrently, drug repositories began to annotate bioactivity data for hundreds of drugs (Liu *et al.* 2007, Wishart *et al.* 2008, Gaulton *et al.* 2012). With the consolidation of large cell line panels, traditional OMICS started to gather all sorts of biological descriptors, from mutations in the genome to protein abundances (Barretina *et al.* 2012, Yang *et al.* 2013). The next generations incorporated global biological responses to small molecules and genetic perturbations (Lamb *et al.* 2006, Seashore-Ludlow *et al.* 2015, Tsherniak *et al.* 2017). Eventually, the accumulation of genomic data enabled the statistical exploration of gene–phenotype associations, leading to the extensive identification of disease-associated genes (Li *et al.* 2012, Piñero *et al.* 2015). All these initiatives are populating biomedical repositories with hundreds of datasets, many of which are part of monumental efforts that still keep providing new releases to date (e.g. Wishart *et al.* 2018, Ghandi *et al.* 2019, Luck *et al.* 2020, Piñero *et al.* 2020). Many of these initiatives are gigantic efforts that take decades to complete and, while first-in-class datasets often offer a wealth of new biological findings, it is important to assess the novelty of subsequent releases to determine the optimal screening strategies (i.e. by focusing the screening on under-explored areas). Thus, it is paramount to have the means to contextualize new data in light of current biomedical knowledge. Indeed, it is a common practice to inspect experimental results based on existing data, as it helps to validate new methodologies and the results, thereby, gaining confidence in the provided insights. Unfortunately, no standard exists for this analysis, which inevitably hampers the fair comparison with existing resources. Besides, these assessments usually use previous releases or analogous datasets as a reference, missing whether similar relationships have been found in orthogonal studies. Here, we present Bioteque Supports (BQsupports), a web tool to systematically quantify the support of novel biological associations, including links between the same type of biological entities (i.e. PPIs or disease–disease comorbidities) and heterogenous relations (i.e. disease-gene associations or drug-cell sensitivities), based on the current biomedical knowledge pre-encoded in the Bioteque (Fernández-Torras *et al.* 2022). BQsupports assesses the similarity of each pair of biomedical entities given by the user within a collection of diverse Bioteque descriptors, providing support scores across various biomedical contexts. This is, BQsupports looks for evidence that potentially backs up the given link between biological entities on the over 800 types of relationships included in the Bioteque (e.g. genes in similar pathways or diseases). Additionally, the tool suggests the type of descriptors, in the form of low-dimensional numerical vectors (i.e. embeddings), which better recapitulate the input pairs, thus providing a suitable framework to predict relationships between entities that the screens might have missed. Overall, by exploring hundreds of descriptors, BQsupports provide support scores for novel experimental observations across a wide variety of biomedical contexts.

## 2 BQsupports description

BQsupports provides biomedical support scores between pairs of biological entities given by the user, from properly connected networks to isolated paired observations. This support derives from biomedical knowledge descriptors (i.e. embeddings) gathered in the Bioteque resource (Fernández-Torras *et al.* 2022), which can assess links between 11 different types of biomedical entities, namely: genes/proteins (GEN), cell lines (CLL), tissues (TIS), small molecule compounds (CPD), diseases (DIS), pharmacological classes (PHC), chemical entities (CHE), pathways (PWY), cellular components (CMP), protein domains (DOM), and molecular functions (MFN). In brief, we collected and harmonized experimentally determined relationships between these biomedical entities (i.e. nodes), which we used to build a biomedical knowledge graph (KG). We then explored the KG in a context-specific manner, using network paths guided through specific entities and relations (i.e. metapaths), describing a rich variety of biomedical contexts, which we condensed in the form of network embeddings. More specifically, we pre-computed over 1000 metapath embeddings, so that a given biological entity can be described in many different ways, depending on its context. For instance, we have different descriptors for a protein accounting for its pattern of interactions, the cells or tissues where it is expressed, the diseases where it is involved or the drugs targeting it. Overall, nodes that are close in a given metapath embedding show similar biomedical properties in that context. Given a set of node pairs covered by the Bioteque, BQsupports automatically identifies the metapaths potentially related to the input data.

**Figure 1.**
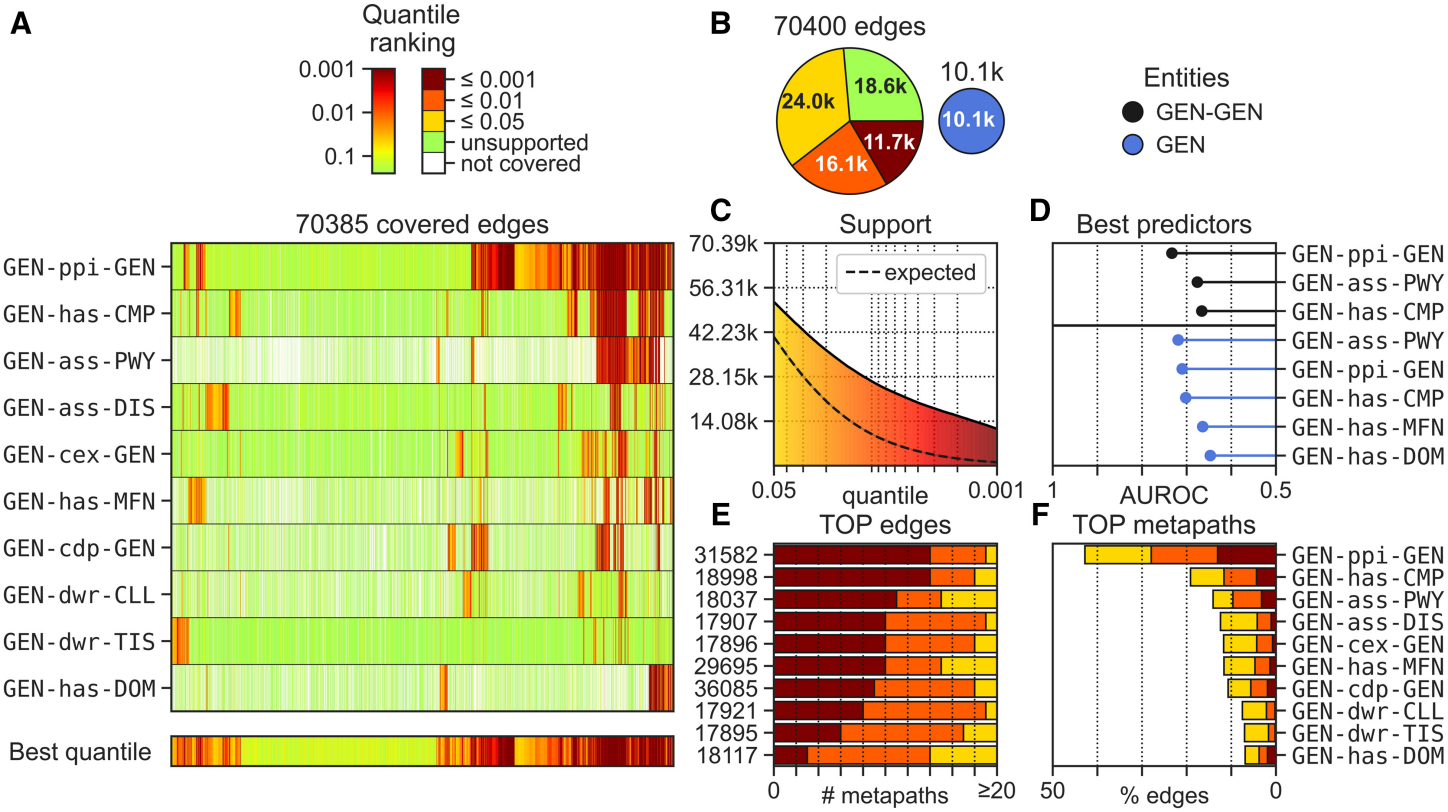
BQsupports analytical canvas for the Bioplex protein–protein interaction data. (a) Quantile ranking (support score) for all the input relationships (*y*-axis) covered by the top 10 most supportive metapaths (*x*-axis). The lower (redder) the quantile rank, the higher the support score. (b) Support scores stratified according to different cutoffs, along with the coverage of the input dataset (minor pie chart). (c) Support of the input dataset across different quantiles and the ‘expected support’ according to the random network permutations. (d) Top metapaths able to predict the input data quantified by the Area Under the ROC (*x*-axis). The top 3 metapaths able to predict the dataset associations from random permutations are shown in black, and the top metapaths able to predict genes of the given dataset having a similar interacting profile (i.e. genes interacting with the same genes) in blue, as detailed in Supplementary Methods. Notice that if a heterogeneous network (i.e. involving different types of entities) is provided, the analysis will be done individually for each entity type and the results will be colored according to the entity color code used in the Bioteque. (e) Top input edges with more accumulated support. The edge network position is shown in the *y*-axis, while the number of metapaths supporting the edge is quantified in the *x*-axis at different quantile rank cutoffs. (f) Ranking of metapaths (*y*-axis) according to the percentage of dataset edges (*x*-axis), they support (stratified at different quantile rank cutoffs).

Then, within the context provided by each metapath space, it measures the cosine distance between the corresponding node descriptors in each pair, and provides an associated support score. These individual, context-related, scores are further aggregated to obtain a single support estimate for the entire dataset. Moreover, the tool automatically runs a network permutation protocol to (i) derive the expected support of the dataset and (ii) detect entity pairs that are significantly close (supported) in a particular biomedical context, which are quantified by an enrichment score. Additionally, BQsupports identifies metapaths able to distinguish the dataset associations from random permutations, thus providing means to prospectively predict associations that might be missing in the input dataset. All the analyses and results for each input data can be downloaded in the form of different tables and they are summarized in a canvas picture. The entire pipeline and the output files and formats are detailed in the Supplementary Methods section. As an illustrative example, Fig. 1 shows the BQsupport results for the Bioplex-III PPI network (Huttlin *et al.* 2021). The heatmap on the left (a) shows the level of support that different metapaths (rows) give to each PPI in the dataset (columns). When considering all the biomedical context descriptors together (last row), we see that over 73% of the interactions are supported by current knowledge (quantile rank ≤ 0.05). In other words, 27% (19.1k) of the PPIs identified in the Bioplex-III network can be considered potentially novel, according to our resource. The expected support suggests that the interactions in this dataset are more backed up by other observations than expected by chance, especially within the stronger (redder) support range. The TOP metapath ranking shows that most of this support (more than 40% of the edges) comes from descriptors based on previously known PPIs (GEN-ppi-GEN), followed by descriptors finding the provided interactions in similar cell compartments (GEN-has-CMP) and pathways (GEN-ass-PWY). Not surprisingly, these three metapaths are the ones suggested by BQsupports to be more suitable for predicting PPIs that the current versions of Bioplex might have missed.

## 3 Concluding remarks

BQsupports offers a means to systematically assess new experimental data with respect to known observations as collected in the Bioteque, providing support scores for each observation in a given dataset while identifying features suitable for downstream predictive tasks. BQsupports is available as a web-based tool, where the user is only asked to (i) provide the dataset associations with proper identifiers, (ii) specify the types of biomedical entities and (iii) optionally tune some parameters (e.g. the number of permuted networks). With default parameters, the whole BQsupports pipeline takes between 1 and 2 h to complete, depending on the number of relationships provided (Supplementary Methods). At the end of the process, BQSupports returns a canvas figure summarizing the results (Fig. 1 is the direct output of the pipeline ran on Bioplex-III) and three table files covering the quantile ranking score for each association-metapath combination, a digested summary counts for each descriptor, and the estimated performance of these metapaths in downstream predictive tasks. Indeed, the provided files allow the user to easily recompute most of the presented analyses according to custom needs (i.e. limiting the support score to a particular set of biomedical contexts or requiring a minimum enrichment score). Also note that all the metapath descriptors can be downloaded from the main Bioteque page (https://bioteque.irbbarcelona.org). Beyond offering an initial assessment of the novelty of new experimental data, we foresee other potential uses for our tool. For instance, the BQsupports pipeline can readily identify the biological contexts and databases that relate two biological entities, providing a starting point for hypothesis generation. This can be interesting in cases where a given relationship is observed but we are unaware of other underlying connections which may add pertinent context (e.g. when a set of drug biomarkers are identified in a screening and want to explore the relation of these biomarkers (GENs) to the potential targets of the drug (CPD-int-GEN or CPD-int-GEN-ppi-GEN) or treatment (CPD-trt-DIS-ass-GEN)).

Additionally, BQsupports can be used at the beginning of a machine learning pipelines to efficiently explore which type of descriptors are more suitable for predicting new associations. That is to say, the training data of the model could first be analyzed by BQsupports and the metapath that can recapitulate better the initial connections can be used as descriptors (i.e. model features) of the given data to train the model. Indeed, we used this strategy to identify Bioteque descriptors recapitulating drug-disease treatment relations which we later used to predict new drug-repurposing opportunities (Fernández-Torras *et al.* 2022). Finally, we would like to highlight that the BQsupports pipeline is constrained to the databases covered by the Bioteque. Biomedicine is an ever-growing field and, despite our efforts to incorporate a major representation of its knowledge, there will still be missing entities, relationships and datasets in our resource, and thus the coverage of the provided results should be interpreted with caution.

## Supplementary Material

btad581_Supplementary_Data
