# Supplementary material for: BQsupports: systematic assessment of the support and novelty of new biomedical associations
Source: Bioinformatics. 2023 Sep 19;39(9):btad581. doi: 10.1093/bioinformatics/btad581 (PMC10521632; doi:10.1093/bioinformatics/btad581)
Supplement: btad581_Supplementary_Data [file btad581_supplementary_data.pdf]

BQsupports: systematic assessment of the support and novelty of new biomedical associations

Adrià Fernández-Torras[1], Martina Locatelli[1], Martino Bertoni[1] and Patrick Aloy[1,2,*]

1. Institute for Research in Biomedicine (IRB Barcelona), The Barcelona Institute of Science and Technology, Barcelona, Catalonia, Spain
2. Institució Catalana de Recerca i Estudis Avançats (ICREA), Barcelona, Catalonia, Spain

* Corresponding author: patrick.aloy@irbbarcelona.org

Supplementary Data

BQsupports coverage and data sources

BQsupports fetches biomedical descriptors from the Bioteque resource (Fernandez-Torras, et al., 2022). At the date of publication, it covers 11 different entities (nodes), allowing for the screening of 11 homogeneous and 110 bipartite entity-entity combinations (edges). Each entity type has its identifier vocabulary, enabling a harmonized data integration. The available entities, vocabularies, and metapath descriptors are specified in the web resource (https://bioteque.irbbarcelona.org).

The data sources used by the tool and the scripts to preprocess them can be obtained from https://bioteque.irbbarcelona.org/sources. Additionally, the universe of metapath-source combinations and the available entities can be downloaded from https://bioteque.irbbarcelona.org/downloads.

Scalability

We tested the tool with tens of different datasets and confirmed that it scales well with hundreds of thousand edges. However, we capped the maximum number of edges to 1M to control the computational resources. With the default number of permutations (20) to estimate the expected level of support, the entire pipeline takes between 1h to 2h to run, depending on the entity types and number of edges (Figure S1, left). However, we observed that the number of permuted networks significantly impacts the computational time. For example, in the Bioplex-III network, moving from 20 to 100 random permutations added two hours of extra computation, while moving from 100 to 1000 added 18h more (Figure S1, right).

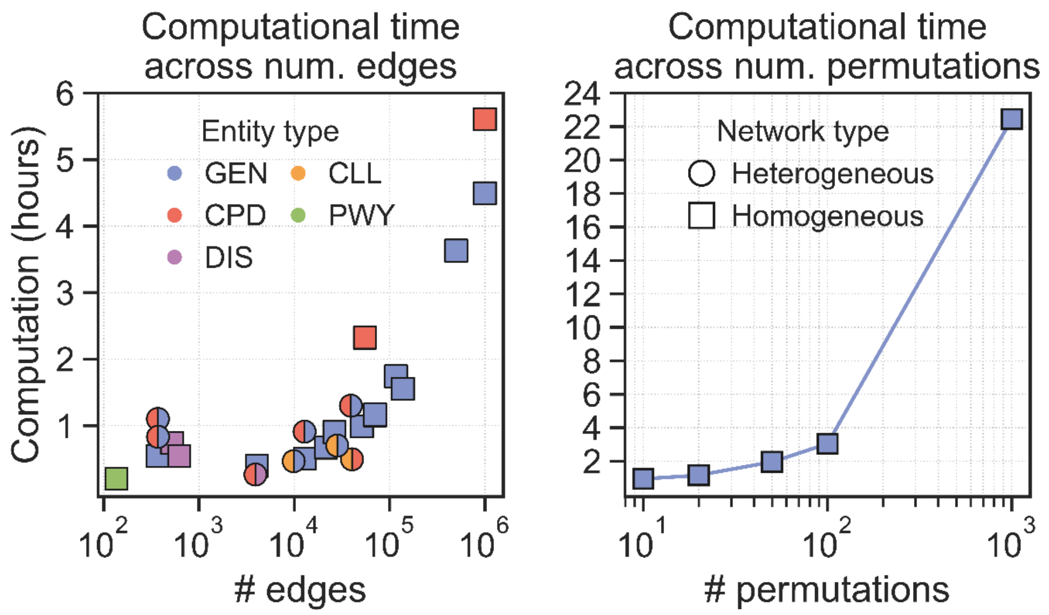

Figure S1. The computational time of the BQsupports pipeline. *Left) Computational time in hours (y-axis) taken to run the full pipeline for different networks of varying size (x-axis) and type (shape and color). Right) Computational time in hours (y-axis) taken to run the BQsupports pipeline on the Bioplex-III network using 10, 20, 50, 100, and 1000 network permutations (x-axis).*

Methodology

Data input and user interface

BQsupports accepts pairs of associated nodes (networks) as input data. Users can provide the data either explicitly to the web or by uploading an edge file. Next, the user has to specify the type of entities provided. When providing homogeneous networks, it is possible to specify whether the associations are undirected (e.g. protein-protein interactions) or directed (kinase-substrate interactions). This will affect the network permutation process (e.g. in a directed kinase-substrate network, random permutations will always produce kinase-substrate pairs). Users can also vary the number of permuted networks from 10 to 1000. By default, the tool uses 20 network permutations, allowing a p-value resolution of 0.05. Note that the number of permuted networks directly impacts the enrichment score, where statistical power increases proportionally to the number of permutations (i.e. enrichment scores tend to be more significant and accurate). However, increasing the number of permuted networks will also affect the computational time of the pipeline (Figure S1, right). Lastly, it is important to note that BQsupports will skip repeated edges, as they can artificially increase the support of the dataset. If the provided network is undirected, the pipeline will sort each edge before removing duplicates. At the end of the process, the final network is provided to the user.

Calculation of support scores

The pipeline starts by listing all the metapaths connecting the entities specified by the user. It considers metapaths of any length available in the Bioteque resource, except for GEN-GEN associations, which are limited to L1 metapaths. BQsupports omits metapaths covering less than 10% of the data.

Once the metapath universe is defined, it computes cosine distances between each provided association in each metapath space and ranks them according to the metapath distance distribution. To obtain these rankings efficiently, only the top 25% closest neighbors (first quartile) for each node are retrieved using FAISS (Johnson, et al., 2017). Accordingly, the node found in the first quartile sets the maximum ranking distance in the metapath. Next, BQsupports transforms rankings into quantiles by dividing them by the number of nodes in the metapath space. Finally, as this process generates two quantiles (i.e. one ranking for each node), it derives an edge-level quantile by keeping the geometric mean of the pair (i.e. the normalized co-rank). This process is repeated independently for each metapath-dataset descriptor in the pre-selected universe.

Calculation of random permutations and enrichment scores

To generate random permutations of the data, we perform *n* random swaps of the network using the BiRewire Bioconductor package (Gobbi, et al., 2022), where *n* is fixed to be ten times the number of edges in the dataset. Then, quantile ranking scores are calculated independently for each network permutation following the pipeline described in the previous section.

Enrichment scores are computed for each metapath and edge-level quantile. More specifically, given a metapath-source descriptor space and a quantile cutoff (tested range between 1 and 0.001), the pipeline first annotates the number of associations in the given dataset that score lower than the given quantile cutoff. Then, it obtains a Fold Change (FC) by dividing this number by the median number of associations obtained from the random permutations. Additionally, it derives an empirical p-value by counting the proportion of permuted networks with equal or more associations than the original dataset. Notice that the resolution of this p-value will depend on the number of permuted networks (e.g. given 20 random permutations, the lowest computable p-value is < 0.05).

Networks covering most of the theoretical possible connections (i.e. dense networks) will be prone to yield permutations overlapping with the input data, consequently diminishing the enrichment. BQsupports will raise a warning when the median overlap of the permutations is higher than 10%. However, while lack of enrichment implies that the degree of support of a given edge is not network specific (i.e. it can be likely obtained by network permutations), it does not invalidate the statistical significance of the quantile ranks obtained for the edge. Thus, it is on users' judgment whether the lack of enrichment

may compromise the results of the analysis or, on the contrary, is not relevant (or even expected) given the goal and/or structure of the data.

Identifying the best metapaths to complete missing node associations

To suggest metapaths suitable for dataset-specific edge prediction, the tool evaluates the capacity of metapath descriptors to distinguish the dataset associations from random permutations by ranking all the associations according to their cosine similarities. In those metapath spaces where ‘real’ edges (i.e. those given by the user) are up-ranked before random permutations, we can assume that the space preserves the structure of the dataset. Thus, the descriptors of this metapath space are likely to embed useful information to predict such type of relationships between the nodes. This is quantified by computing the Area Under the Receiver Operating Characteristic (AUROC) curve between the user edges and 10 random permutations. To prevent an association from being counted as a positive and negative instance simultaneously, the pipeline generates new random permuted networks without allowing them to overlap with the data provided by the user. At the end of the process, BQsupports provides the AUROC average across the 10 permutations together with the universe of each metapath and the covered portion of the input dataset. Note that the covered data represent the applicability domain of the computed AUROC.

Additionally, the pipeline also looks for metapath descriptors that, while not directly preserving the associations provided by the user, retain the neighborhood similarity of the nodes. In other words, BQsupports first identifies nodes having similar interactions in the network supplied by the user, and then tries to find metapath spaces in which these nodes are similar. To this aim, the pipeline first builds a new network by linking the input nodes between them provided that they belong to the same entity type and have in common a significant number of associations (i.e. they are similar interactors). More specifically, this new network is created by (i) representing each node with a binary vector annotating their interactions (i.e. the adjacency matrix), (ii) calculating term frequency-inverse document frequency (TF-IDF) values between the vectors and (iii) keeping the top 3 neighbors with highest TF-IDF similarity for each node. As a result, a new homogenous network is obtained, whose edges capture the most similar interacting nodes from the network provided by the user. Next, the pipeline lists a new metapath universe for this network and computes the recapitulation (AUROC) scores of these metapaths, using the same approach described above. As a result, BQsupports identifies metapaths that keep the similarities between the nodes. Note that metapaths identified in this analysis may be different from those found to yield the highest prediction potential, even for homogeneous networks. For instance, in the example provided in Figure 1, the suggested entity predictors include two metapaths, namely GEN-has-MFN and GEN-has-DOM, that were not covered by the previous analysis. This would indicate that genes having similar molecular functions (GEN-has-MFN) or sharing protein domains (GEN-has-DOM) may

not necessarily interact with each other but tend to interact with the same proteins, to some extent.

Output canvas generation

1. Heatmap. To generate the heatmap matrix, BQsupports first aggregates the scores by keeping the best quantile ranking among the sources belonging to the same metapath, obtaining a unique score per metapath. Then, it ranks metapaths according to the number of interactions they support with a quantile lower than 0.05, selecting the top 10 for the heatmap. Additionally, it provides the best quantile across all the screened metapaths in the last row. Associations not covered by a given metapath are left blank. Note that quantile scores are capped at 0.25 (1st quartile), as higher quantiles are ignored by the pipeline.

2. Pie charts. Support scores for each dataset association are aggregated by selecting the best score across metapaths. Next, they are stratified into four groups according to their quantile: *≤0.001, ≤0.01, ≤0.05,* and *unsupported* (quantile > 0.05). The pie chart reports the counts of each group, together with those not covered by the resource (if any). Additionally, a minor pie chart depicts the fraction of nodes covered for each entity, colored according to the color code used in the Bioteque resource.

3. Dataset support. The total number of supported associations in the dataset is reported across the range of significant quantile rankings (from 0.05 to 0.001). Additionally, BQsupports annotates the mean and standard deviation achieved with permuted networks (dashed line), providing the expected supportiveness according to the dataset's applicability domain (universe).

4. Edge and metapath ranking. The top 10 most supported edges and supportive metapaths are ranked according to the number of metapaths (or edges) with a quantile lower than 0.05. BQsupports uses the index provided in the original network (starting the count from 1) to label the edges in the plot (shown on the y-axis).

5. Best metapath predictors. The canvas shows the top 3 metapath for each tested network (i.e. the one provided by the user and each entity-entity similarity network generated by BQsupports). In this case, the reported scores are not aggregated by metapath and they correspond to a specific metapath-source combination. Furthermore, the tool only shows significant and relevant metapath-source combinations, those whose average AUROC value (after subtracting their standard deviation) is higher than 0.6 and cover at least 20% of the dataset.

Output files

The pipeline outputs the following files:

- *canvas.png*: PNG figure summarizing the results of the analysis.
- README.txt: Text file with the legend for the outputted files and figures.
- supports.tsv: Tab-separated file providing the quantile ranking (support), cosine distance, z-scored cosine distance, and enrichment scores (with its corresponding p-value as detailed in the 'Random permuted networks and enrichment score inference' section) for each edge in each metapath-source. Enrichment scores are not computed for datasets with less than 50 edges.
- metapath_counts.tsv: Tab-separated file providing the number of edges supported by each metapath-dataset at different support score cutoffs.
- edge_recapitulation.tsv: Tab-separated file providing the recapitulation score (AUROCs) for each metapath assessed as potential predictor of the given network. The file also provides the coverage of the dataset and the size of the metapath universe. This file is not provided if the given network has less than 50 edges.
- XXX_neighbours_recapitulation.tsv: Tab-separated file providing the recapitulation score (AUROCs) for each metapath assessed as potential predictor of similar interactors in the network. The file also provides the coverage of the dataset and the size of the metapath universe. An individual file is outputted for each entity in the dataset (annotated in the prefix of the file). This file is not provided if the interactor network computed by BQsupports has less than 50 edges.
- WARNINGS.txt (optional): Text file with the warnings that happened during the processing of the network (e.g. steps that were skipped due to lack of data, if any).

Supplementary references

Fernandez-Torras, A., *et al.* Integrating and formatting biomedical data as pre-calculated knowledge graph embeddings in the Bioteque. *Nat Commun* 2022;13(1):5304.

Gobbi, A., *et al.* 2022. BiRewire: High-performing routines for the randomization of a bipartite graph (or a binary event matrix), undirected and directed signed graph preserving degree distribution (or marginal totals). Release R package version 3.28.0. https://bioconductor.org/packages/BiRewire

Johnson, J., Douze, M. and Jégou, H. Billion-scale similarity search with GPUs. *arXiv [cs.CV]* 2017.
